# Supplementary material for: Histone H2B.8 compacts flowering plant sperm through chromatin phase separation
Source: Nature. 2022 Nov 2;611(7936):614–22. doi: 10.1038/s41586-022-05386-6 (PMC9668745; doi:10.1038/s41586-022-05386-6)
Supplement: Supplementary file 1 — This file contains Supplementary Figs. 1 and 2 and Supplementary Tables 2–7 (see separate file for Supplementary Table 1). [file 41586_2022_5386_MOESM1_ESM.pdf]

nature portfolio

https://doi.org/10.1038/s41586-022-05386-6

Supplementary information

Histone H2B.8 compacts flowering plant sperm through chromatin phase separation

In the format provided by the authors and unedited

Extended Data Figure 1a - Rep1 H3K9me2

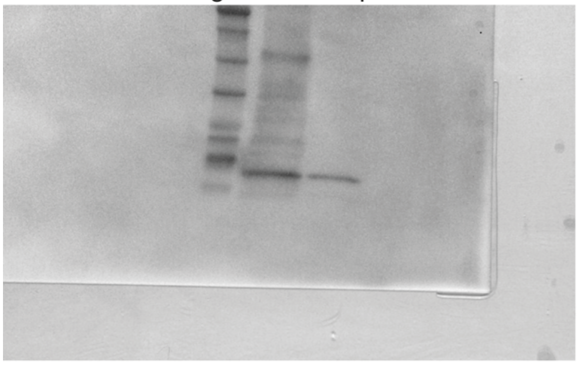

Extended Data Figure 1a - Rep1 H3

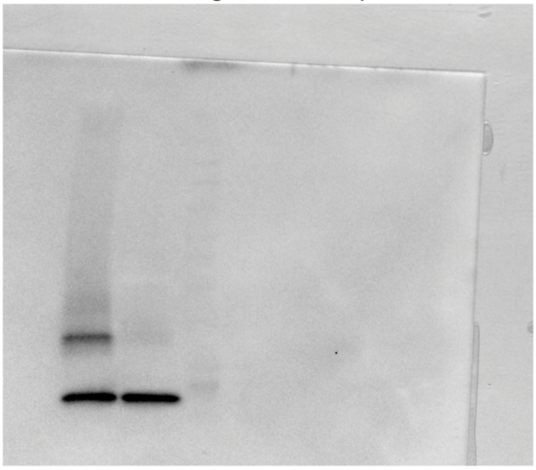

Extended Data Figure 1a - Rep2 H3K9me2

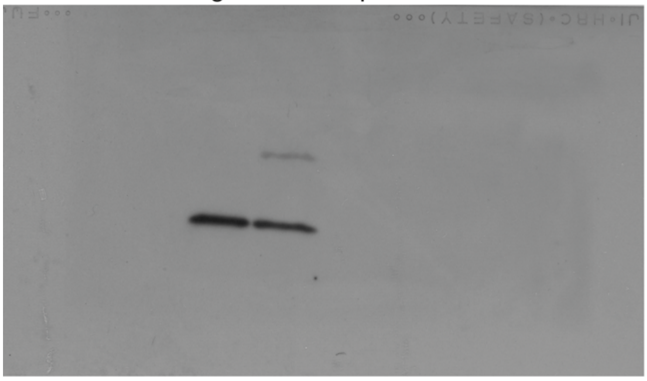

Extended Data Figure 1a - Rep2 H3

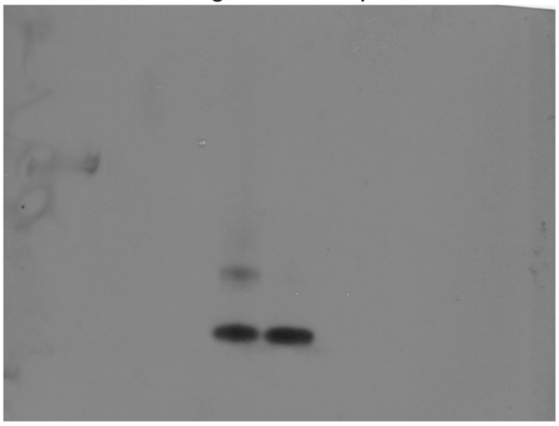

Extended Data Figure 3c - H2B.2 and H2B.8

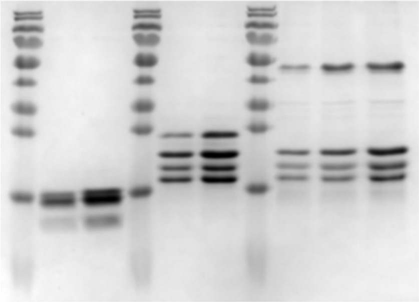

Extended Data Figure 3c - H2B.8ΔIDR

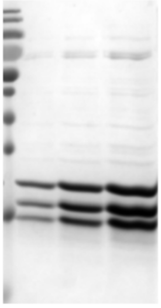

Extended Data Figure 3c - H2B.8-scrambledIDR

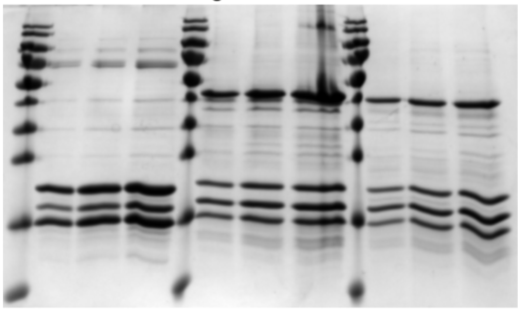

Supplementary Figure 1. Source data for blots or gels in Extended Data Figure 1a and Extended Data Figure 3c.

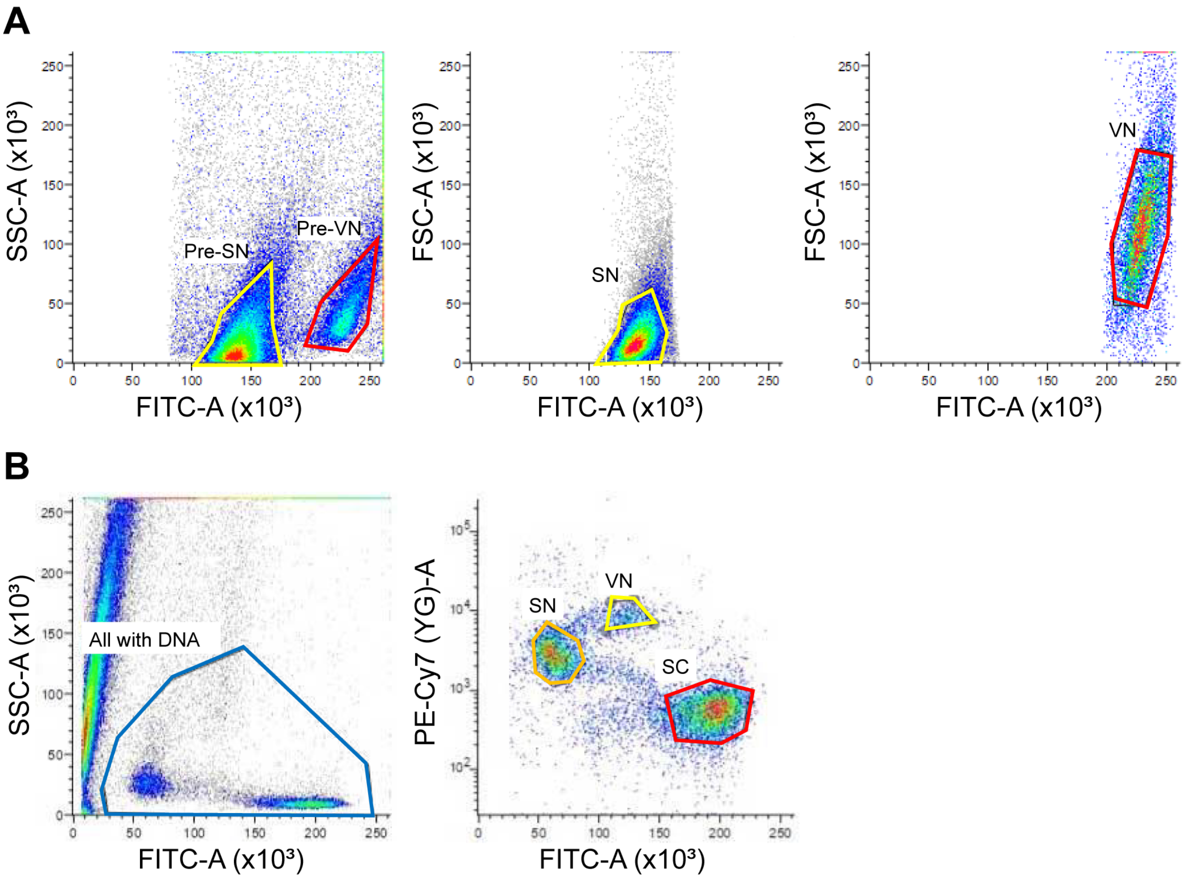

Supplementary Figure 2. Example FACS scatterplots showing **(A)** the gating strategy to isolate sperm (SN) and vegetative (VN) nuclei by SYBR Green intensity (FITC-A) and side scatter area (SSC-A) and then by forward scatter area (FSC-A) and **(B)** the gating used to purify sperm cells (SC) from pollen by first gating for all events with DNA by SYBR Green intensity (FITC-A) and side-scatter (SSC-A) and then against SYTOX Orange staining (PE-Cy7 (YG)-A).

Supplementary Table 1. List of H2B.8 homologs identified in flowering plant species.

File attached.

Supplementary Table 2. Multivariate linear regression modelling of H2B.8. The table lists the regression weight for each variable in the multivariate linear regression models (MLR1 and MLR2) of H2B.8. The formula and adjusted R^2 for both models are shown (P-value < 0.001, F-statistic, for all variables in both models). Additionally, for each variable, a separate model was made to predict H2B.8 and the adjusted R^2 value is listed.

**MLR1**	**H3K4me3**	**Log_{10} (RPKM+1)**	**GC**	**H3K9me2**	**H1.1**	**H1.2**	**H2A.W**
Regression weight	-0.276	-0.334	-1.654	0.017	-0.176	-0.129	0.017
Adjusted R^2	0.360	0.342	0.205	0.021	0.110	0.124	0.013
**MLR1**	**H2A.Z**	**H3.1**	**H3.3**	**H3K27ac**	**H3K27me1**	**H3K27me3**	**mCG**
Regression weight	0.204	-0.154	0.240	0.079	0.122	0.088	0.231
Adjusted R^2	0.001	0.016	0.012	0.077	0.033	0.022	0.002
MLR1: H2B.8 = 1 - 0.276×H3K4me3 - 0.334×Log10(RPKM+1) - 1.654×GC + 0.017×H3K9me2 - 0.176×H1.1 - 0.129×H1.2 + 0.017×H2A.W + 0.204×H2A.Z - 0.154×H3.1 + 0.240×H3.3 + 0.079×H3K27ac + 0.122×H3K27me1 + 0.088×H3K27me3 + 0.231×mCG (Adjusted R^2 = 0.567)							
**MLR2**	**gene TPM>5**	**gene TPM 1-5**	**gene TPM 0-1**	**euTE**	**hetTE**	**intergenic**	
Regression weight	-0.933	-0.791	-0.097	0.613	-0.190	0.666	
Adjusted R^2	0.183	0.091	0.0001	0.061	0.0008	0.209	
MLR2: H2B.8 = - 0.074 - 0.933×gene (TPM>5) - 0.791×gene (TPM 1-5) - 0.097×gene (TPM 0-1) + 0.613×euTE - 0.190×hetTE + 0.666×intergenic (Adjusted R^2 = 0.422)							

Supplementary Table 3. *h2b.8* transmission data. *h2b.8*+/-, heterozygous *h2b.8* mutant; *P* value, Fisher's exact test. Results shown in the first two rows were obtained from one reciprocal crossing experiment performed by one researcher, and those from the third row were obtained from a separate experiment performed by an independent researcher.

Maternal parent	Paternal parent	Total F1 progeny	WT F1 progeny	*h2b.8*+/- F1 progeny	Ratio (WT: *h2b.8*+/-)	*h2b.8* transmission (%)	*P* value
Wild type	*h2b.8* +/-	575	333	242	1:0.73	42.09	0.0078
h2b.8 +/-	Wild type	574	278	296	1:1.06	51.57	0.6367
Wild type	*h2b.8* +/-	743	436	307	1:0.70	41.32	0.0009

Supplementary Table 4. *In vitro* germination assay of wild-type and *h2b.8* mutant pollen.

Rep	Genotype	Elongated (%)	Germinated (%)	Not germinated (%)
1	WT	85.37	5.11	9.52
2	WT	80.9	4.33	14.77
3	WT	81.74	6.27	11.99
4	WT	80.49	7.38	12.13
5	WT	85.51	3.25	11.24
6	WT	85.73	2.07	12.2
Average	WT	83.29	4.74	11.97
1	*h2b.8*	85.4	3.32	11.28
2	*h2b.8*	86.98	2.22	10.79
3	*h2b.8*	86.36	2.91	10.73
4	*h2b.8*	77.08	6.25	16.67
5	*h2b.8*	85.81	1.92	12.28
6	*h2b.8*	82.87	3.69	13.44
Average	*h2b.8*	84.08	3.39	12.53

Supplementary Table 5. Segregation among progeny from self-pollinated *h2b.8* heterozygous mutant plants.

Selfed F1 plant	F2 # WT	F2 # *h2b.8* +/-	F2 # *h2b.8* -/-	Total F2 #
1	97	174	85	356
2	103	190	88	381
3	93	193	97	383
4	64	192	86	342
total	357	749	356	1462

Supplementary Table 6. List of primers used in this study.

Primer name	Sequence (5' - 3')	Purpose	Construct
**H2B.8 CRISPR sgRNA**			
TB201	TGTGGTCTCAATTGACAATCAAAGTAACCGTCAGTTTAAGAGCTATGCTGGAA	H2B.8 sgRNA 1	pICSL4723 with 4 H2B.8 sgRNA via pICH47751
TB202	TGTGGTCTCAATTGAAGTAACCGTCACGGAAGAGTTTAAGAGCTATGCTGGAA	H2B.8 sgRNA 2	pICSL4723 with 4 H2B.8 sgRNA via pICH47761
TB203	TGTGGTCTCAATTGGGATCTCCTTCTTCCGTGAGTTTAAGAGCTATGCTGGAA	H2B.8 sgRNA 3	pICSL4723 with 4 H2B.8 sgRNA via pICH47772
TB204	TGTGGTCTCAATTGCTGTCTCGGTGATCACACAGTTTAAGAGCTATGCTGGAA	H2B.8 sgRNA 4	pICSL4723 with 4 H2B.8 sgRNA via pICH47781
**H2B.8 CRISPR screening**			
SH335	CCACCGCGTAGTAGACAG	H2B.8 forward primer	N/A
SH336	AAGCGGGAGTTTCCGGTG	H2B.8 reverse primer	N/A
SH337	GCTAAGGTATTCGAACGAC	H2B.8 CRISPR sequencing primer	N/A
TB296	AGGTCGTCGTCGTAGGTGTC	Cas9 forward primer	N/A
TB297	GTGCAGACCTACAACCAGCT	Cas9 reverse primer	N/A
***h2b.8-1* and *h2b.8-2* genotyping**			

TB298	AGTAGACAGTTAATCACCAATGCTAAGGTA	*h2b.8-1* dCAPS genotyping forward nested PCR	N/A
TB299	TGATCACACACGGATCTCCTTCCTCCGTGA	*h2b.8-1* dCAPS genotyping reverse nested PCR	N/A
TB293	AGTAGACAGTTAATCACCAATGCTAAGGTA	*h2b.8-1* dCAPS genotyping forward	N/A
TB294	TGATCACACACGGATCTCCTTCCTCCGTGA	*h2b.8-1* dCAPS genotyping reverse	N/A
TB335	GTGAAAGGGTGATCGTGGTG	*h2b.8-2* genotyping forward	N/A
TB336	TGGCGGGAGATGAGTATAGG	*h2b.8-2* genotyping reverse	N/A
**Reporter and ectopic H2B.8 cloning (Multisite Gateway cloning system)**			
TB137	GGGGACAACTTTGTATAGAAAAGTTGATCAGTGGAGGATGACATGGC	H2B.8 promoter cloning forward with attB4 site	*pH2B.8::H2B.8-eGFP* / *pH2B.8::H2B.8-Myc*
TB138	GGGGACTGCTTTTTTGTACAAACTTGATTCTTCGTTAGAAATAACCG	H2B.8 promoter cloning reverse with attB1r site	*pH2B.8::H2B.8-eGFP* / *pH2B.8::H2B.8-Myc*
TB139	GGGGACAAGTTTGTACAAAAAAGCAGGCTATGGCGCCGAGAAAACCAAAGGT	H2B.8 cloning forward with attB1 site	*pH2B.8::H2B.8-eGFP* / *pH2B.8::H2B.8-Myc* / *p35S::H2B.8-eGFP*
TB140	GGGGACCACTTTGTACAAGAAAGCTGGGTAACGTTTCCTACTATCATAACCA	H2B.8 cloning reverse with attB2 site	*pH2B.8::H2B.8-eGFP* / *pH2B.8::H2B.8-Myc* / *p35S::H2B.8-eGFP*

**H2B.8ΔIDR and H2B.2 cloning (In-Fusion cloning system)**			
SH469	TATGACCATGATTACGAATTATCAGTGGAGGATGACATGGCG	H2B.8ΔIDR overlapping forward N-terminus	*p35S::H2B.8ΔIDR-YFP / pH2B.8::H2B.8ΔIDR-eGFP*
SH475	TCAAAGTGCCGACGGTTACTTTGATTGTCTCTTCC	H2B.8ΔIDR overlapping reverse N-terminus	*p35S::H2B.8ΔIDR-YFP / pH2B.8::H2B.8ΔIDR-eGFP*
SH476	AGTAACCGTCGGCACTTTGAAGAAAACAGATAAGG	H2B.8ΔIDR forward C-terminus	*p35S::H2B.8ΔIDR-YFP / pH2B.8::H2B.8ΔIDR-eGFP*
SH474	CTCGCCCTTGCTCACGGATCCCAGATCTTCCTCAGAGATGAGC	H2B.8ΔIDR reverse C-terminus	*p35S::H2B.8ΔIDR-YFP / pH2B.8::H2B.8ΔIDR-eGFP*
TB333	TCTATCTCTCTCGAGGTACCATGGCGAAGGCAGATAAGAAA	H2B.2 cloning forward	*p35S::H2B.2-YFP*
TB335	TGTCGACTCCGAATTCAGAACTCGTAAACTTCGTAACCGC	H2B.2 cloning reverse	*p35S::H2B.2-YFP*
**Chimeric H2B.8 construct cloning (Multisite Gateway cloning system)**			
PSH295	GGGGACCACTTTGTACAAGAAAGCTGGGTAAGAACTCGTAAACTTCGTAACAGCTTTAGAGCCTTC	*H2B.8* with N234K substitution	*pH2B.8::H2B.8-N234K-Myc*
**H2B.8 IDR swap construct cloning (In-Fusion cloning system)**			
PSZ644	ACAAATCTATCTCTCTCGAGGTACCATGGCGCCGAGAAAACCAAAG	H2B.8-EWSR1 overlapping forward N-terminus	*p35S::H2B.8-EWSR1-IDR-eGFP*

PSZ650	CCGTGGACGCGACGGTTACTT TGATTGTCTCTTCC	H2B.8-EWSR1 overlapping reverse N-terminus	*p35S::H2B.8-EWSR1-IDR -eGFP*
PSZ651	AGTAACCGTCGCGTCCACGGA TTACAGTACC	H2B.8-EWSR1 overlapping forward EWSR1-IDR	*p35S::H2B.8-EWSR1-IDR -eGFP*
PSZ652	TCAAAGTGCCCTGCCCATAAA CACCCATGC	H2B.8-EWSR1 overlapping reverse EWSR1-IDR	*p35S::H2B.8-EWSR1-IDR -eGFP*
PSZ653	TTATGGGCAGGGCACTTTGAA GAAAACAGATAAGG	H2B.8-EWSR1 overlapping forward C-terminus	*p35S::H2B.8-EWSR1-IDR -eGFP*
PSZ645	ACTAGTGTCGACTCCGAATTC ACGTTTCCTACTATCATAACCA ACG	H2B.8-EWSR1 overlapping reverse C-terminus	*p35S::H2B.8-EWSR1-eGFP*
PSZ644	ACAAATCTATCTCTCTCGAGGT ACCATGGCGCCGAGAAAACCA AAG	H2B.8-TAF15 overlapping forward N-terminus	*p35S::H2B.8-TAF15-IDR -eGFP*
PSZ666	CAGAATCCGAGACGGTTACTT TGATTGTCTCTTCC	H2B.8-TAF15 overlapping reverse N-terminus	*p35S::H2B.8-TAF15-IDR -eGFP*
PSZ667	AGTAACCGTCTCGGATTCTGG AAGTTACGG	H2B.8-TAF15 overlapping forward TAF15-IDR	*p35S::H2B.8-TAF15-IDR -eGFP*
PSZ668	TCAAAGTGCCTTGTGAATGAT AGGACTGCTGG	H2B.8-TAF15 overlapping reverse TAF15-IDR	*p35S::H2B.8-TAF15-IDR -eGFP*

PSZ669	TCATTCACAAGGCACTTTGAAGAAAACAGATAAGG	H2B.8-TAF15 overlapping forward C-terminus	*p35S::H2B.8-TAF15-IDR-eGFP*
PSZ645	ACTAGTGTCGACTCCGAATTCACGTTTCCTACTATCATAACCAACG	H2B.8-TAF15 overlapping reverse C-terminus	*p35S::H2B.8-TAF15-IDR-eGFP*
PSZ690	TCTATCTCTCTCGAGGTACCATGGCTCCTAGAAAGCCTAAGGT	H2B.8-scrambledIDR forward	*p35S::H2B.8-scrambledIDR-eGFP*
PSZ691	TGTCGACTCCGAATTCTCTCTTTCTAGAATCATATCCAACA	H2B.8-scrambledIDR reverse	*p35S::H2B.8-scrambledIDR-eGFP*

Supplementary Table 7. List of *P*-values associated with statistical tests in this study.

Figure	Group 1	Group 2	P value
Fig. 1b	GN	Leaf	0
	Sperm	Leaf	0
	Sperm	GN	0
Fig. 2a	*h2b.8-1*	WT	0
	h2b.8-2	WT	0
	pH2B.8::H2B.8-eGFP h2b.8-1	WT	0.8040364
	pH2B.8::H2B.8-Myc h2b.8-1	WT	0.7102341
	pH2B.8::H2B.8ΔIDR-eGFP h2b.8-1	WT	0
	pH2B.8::H2B.8-N234K-Myc h2b.8-1	WT	0.5549251
	h2b.8-2	*h2b.8-1*	0.4003808
	pH2B.8::H2B.8-eGFP h2b.8-1	*h2b.8-1*	0
	pH2B.8::H2B.8-Myc h2b.8-1	*h2b.8-1*	0
	pH2B.8::H2B.8ΔIDR-eGFP h2b.8-1	*h2b.8-1*	0.9980652
	pH2B.8::H2B.8-N234K-Myc h2b.8-1	*h2b.8-1*	0
	pH2B.8::H2B.8-eGFP h2b.8-1	*h2b.8-2*	0
	pH2B.8::H2B.8-Myc h2b.8-1	*h2b.8-2*	0
	pH2B.8::H2B.8ΔIDR-eGFP h2b.8-1	*h2b.8-2*	0.9233628
	pH2B.8::H2B.8-N234K-Myc h2b.8-1	*h2b.8-2*	0
	pH2B.8::H2B.8-Myc h2b.8-1	*pH2B.8::H2B.8-eGFP h2b.8-1*	0.9999862
	pH2B.8::H2B.8ΔIDR-eGFP h2b.8-1	*pH2B.8::H2B.8-eGFP h2b.8-1*	0
	pH2B.8::H2B.8-N234K-Myc h2b.8-1	*pH2B.8::H2B.8-eGFP h2b.8-1*	0.9993517
	pH2B.8::H2B.8ΔIDR-eGFP h2b.8-1	*pH2B.8::H2B.8-Myc h2b.8-1*	0
	pH2B.8::H2B.8-N234K-Myc h2b.8-1	*pH2B.8::H2B.8-Myc h2b.8-1*	0.9999006
	pH2B.8::H2B.8-N234K-Myc h2b.8-1	*pH2B.8::H2B.8ΔIDR-eGFP h2b.8-1*	0.92866346

Fig. 2d	*p35S::H2B.8-eGFP #3*	WT	0.002157
	p35S::H2B.8-eGFP #5	WT	0.0003166
	p35S::H2B.8ΔIDR-YFP #2	WT	0.9870122
	p35S::H2B.8ΔIDR-YFP #5	WT	0.9987245
	p35S::H2B.2-YFP #8	WT	0.9890218
	p35S::H2B.2-YFP #9	WT	0.9999989
	p35S::H2B.8-eGFP #5	*p35S::H2B.8-eGFP #3*	0.9993084
	p35S::H2B.8ΔIDR-YFP #2	*p35S::H2B.8-eGFP #3*	0.0268405
	p35S::H2B.8ΔIDR-YFP #5	*p35S::H2B.8-eGFP #3*	0.012598
	p35S::H2B.2-YFP #8	*p35S::H2B.8-eGFP #3*	0.0235015
	p35S::H2B.2-YFP #9	*p35S::H2B.8-eGFP #3*	0.0011189
	p35S::H2B.8ΔIDR-YFP #2	*p35S::H2B.8-eGFP #5*	0.0054939
	p35S::H2B.8ΔIDR-YFP #5	*p35S::H2B.8-eGFP #5*	0.0023178
	p35S::H2B.2-YFP #8	*p35S::H2B.8-eGFP #5*	0.0046637
	p35S::H2B.2-YFP #9	*p35S::H2B.8-eGFP #5*	0.0001511
	p35S::H2B.8ΔIDR-YFP #5	*p35S::H2B.8ΔIDR-YFP #2*	0.9999712
	p35S::H2B.2-YFP #8	*p35S::H2B.8ΔIDR-YFP #2*	1
	p35S::H2B.2-YFP #9	*p35S::H2B.8ΔIDR-YFP #2*	0.9666131
	p35S::H2B.2-YFP #8	*p35S::H2B.8ΔIDR-YFP #5*	0.9999847
	p35S::H2B.2-YFP #9	*p35S::H2B.8ΔIDR-YFP #5*	0.9943575
	p35S::H2B.2-YFP #9	*p35S::H2B.2-YFP #8*	0.970609
Fig. 5c	No change	Down	0
	Up	Down	0
	Up	No change	0
Fig. 5e	WT sperm	Generative nucleus	0
	h2b.8 sperm	Generative nucleus	0.1425682
	pH2B.8::H2B.8ΔIDR-eGFP h2b.8-1	Generative nucleus	0.1395767

	h2b.8 sperm	WT sperm	0.000001
	pH2B.8::H2B.8ΔIDR-eGFP h2b.8-1	WT sperm	0.0000003
	pH2B.8::H2B.8ΔIDR-eGFP h2b.8-1	*h2b.8* sperm	0.9998871
Fig. 5f	WT	*p35S::H2B.8-eGFP*	0
Extended Data Fig. 2b	WT sperm	Generative nucleus	0
	h2b.8-1 sperm	Generative nucleus	0
	h2b.8-2 sperm	Generative nucleus	0
	h2b.8-1 sperm	WT sperm	0
	h2b.8-2 sperm	WT sperm	0
	h2b.8-2 sperm	*h2b.8-1* sperm	0.4120998
Extended Data Fig. 2d	WT	*h2b.8*	3.64E-07
Extended Data Fig. 2e	WT	*h2b.8*	6.44E-14
Extended Data Fig. 2h	Small (left plot)	Large (left plot)	0.00002294
	Small (middle plot)	Large (middle plot)	0.000052222
	Small (right plot)	Large (right plot)	0.6789
Extended Data Fig. 3h	*p35S::H2B.8-eGFP*	WT	0.028352

p35S::H2B.8-scrambledIDR-eGFP	WT	0.0181214
p35S::H2B.8-EWSR1-IDR-eGFP	WT	0.0299781
p35S::H2B.8-TAF15-IDR-eGFP	WT	0.0306313
p35S::H2B.8-scrambledIDR-eGFP	*p35S::H2B.8-eGFP*	0.9999277
p35S::H2B.8-EWSR1-IDR-eGFP	*p35S::H2B.8-eGFP*	0.9967316
p35S::H2B.8-TAF15-IDR-eGFP	*p35S::H2B.8-eGFP*	0.9966074
p35S::H2B.8-EWSR1-IDR-eGFP	*p35S::H2B.8-scrambledIDR-eGFP*	0.9886906
p35S::H2B.8-TAF15-IDR-eGFP	*p35S::H2B.8-scrambledIDR-eGFP*	0.9883868
p35S::H2B.8-TAF15-IDR-eGFP	*p35S::H2B.8-EWSR1-IDR-eGFP*	1
