# Supplementary material for: Histone H2B.8 compacts flowering plant sperm through chromatin phase separation
Source: Nature. 2022 Nov 2;611(7936):614–22. doi: 10.1038/s41586-022-05386-6 (PMC9668745; doi:10.1038/s41586-022-05386-6)
Supplement: Supplementary file 2 — Reporting Summary [file 41586_2022_5386_MOESM2_ESM.pdf]

nature portfolio

Corresponding author(s): Xiaoqi Feng, Pilong Li

Last updated by author(s): Aug 19, 2022

Reporting Summary

Nature Portfolio wishes to improve the reproducibility of the work that we publish. This form provides structure for consistency and transparency in reporting. For further information on Nature Portfolio policies, see our Editorial Policies and the Editorial Policy Checklist.

Statistics

For all statistical analyses, confirm that the following items are present in the figure legend, table legend, main text, or Methods section.

n/a	Confirmed	
☐	☒	The exact sample size (*n*) for each experimental group/condition, given as a discrete number and unit of measurement
☐	☒	A statement on whether measurements were taken from distinct samples or whether the same sample was measured repeatedly
☐	☒	The statistical test(s) used AND whether they are one- or two-sided<br>*Only common tests should be described solely by name; describe more complex techniques in the Methods section.*
☐	☒	A description of all covariates tested
☐	☒	A description of any assumptions or corrections, such as tests of normality and adjustment for multiple comparisons
☐	☒	A full description of the statistical parameters including central tendency (e.g. means) or other basic estimates (e.g. regression coefficient) AND variation (e.g. standard deviation) or associated estimates of uncertainty (e.g. confidence intervals)
☐	☒	For null hypothesis testing, the test statistic (e.g. *F*, *t*, *r*) with confidence intervals, effect sizes, degrees of freedom and *P* value noted<br>*Give P values as exact values whenever suitable.*
☒	☐	For Bayesian analysis, information on the choice of priors and Markov chain Monte Carlo settings
☒	☐	For hierarchical and complex designs, identification of the appropriate level for tests and full reporting of outcomes
☐	☒	Estimates of effect sizes (e.g. Cohen's *d*, Pearson's *r*), indicating how they were calculated

Our web collection on statistics for biologists contains articles on many of the points above.

Software and code

Policy information about availability of computer code

Data collection	Nuclei and cell sorting was performed using a BD FACSMelody™ Cell Sorter with BD FACSChorus™ software version 1.3.
Data analysis	ChIP-seq reads were mapped using Bowtie2-2.3.4.1 and analyzed using deepTools-3.1.1 and BEDtools-2.28.0. RNA-seq reads were mapped using TopHat-2.0.10. Kallisto-0.43.0 and Sleuth-0.30.0 were used to obtain TPM and q-values, respectively. Hi-C data was mapped using the HiC-Pro-2.11.1 pipeline and analyzed using FAN-C-0.9.8. Downloaded bisulfite-seq reads were processed using TrimGalore-0.4.1, mapped using Bismark-0.22.2 and methylation was called using MethylDackel-0.5.2.

For manuscripts utilizing custom algorithms or software that are central to the research but not yet described in published literature, software must be made available to editors and reviewers. We strongly encourage code deposition in a community repository (e.g. GitHub). See the Nature Portfolio guidelines for submitting code & software for further information.

Data

Policy information about availability of data

All manuscripts must include a data availability statement. This statement should provide the following information, where applicable:

- Accession codes, unique identifiers, or web links for publicly available datasets
- A description of any restrictions on data availability
- For clinical datasets or third party data, please ensure that the statement adheres to our policy

Sequencing data generated in this study (ChIP-seq, RNA-seq and Hi-C) have been deposited in the Gene Expression Omnibus (GEO) under accession no. GSE161366.

All remaining data are in the main paper or the supplementary materials. Further information and requests for resources, reagents or code should be directed to Xiaoqi Feng (xiaoqi.feng@jic.ac.uk) and Pilong Li (pilongli@mail.tsinghua.edu.cn).

Human research participants

Policy information about studies involving human research participants and Sex and Gender in Research.

Reporting on sex and gender	*Use the terms sex (biological attribute) and gender (shaped by social and cultural circumstances) carefully in order to avoid confusing both terms. Indicate if findings apply to only one sex or gender; describe whether sex and gender were considered in study design whether sex and/or gender was determined based on self-reporting or assigned and methods used. Provide in the source data disaggregated sex and gender data where this information has been collected, and consent has been obtained for sharing of individual-level data; provide overall numbers in this Reporting Summary. Please state if this information has not been collected. Report sex- and gender-based analyses where performed, justify reasons for lack of sex- and gender-based analysis.*
Population characteristics	*Describe the covariate-relevant population characteristics of the human research participants (e.g. age, genotypic information, past and current diagnosis and treatment categories). If you filled out the behavioural & social sciences study design questions and have nothing to add here, write "See above."*
Recruitment	*Describe how participants were recruited. Outline any potential self-selection bias or other biases that may be present and how these are likely to impact results.*
Ethics oversight	*Identify the organization(s) that approved the study protocol.*

Note that full information on the approval of the study protocol must also be provided in the manuscript.

Field-specific reporting

Please select the one below that is the best fit for your research. If you are not sure, read the appropriate sections before making your selection.

☒ Life sciences ☐ Behavioural & social sciences ☐ Ecological, evolutionary & environmental sciences

For a reference copy of the document with all sections, see nature.com/documents/nr-reporting-summary-flat.pdf

Life sciences study design

All studies must disclose on these points even when the disclosure is negative.

Sample size	No sample-size calculations were performed in this study. Sample sizes were chosen to be representative of the data distribution, similar to previous studies and more than sufficient to calculate statistical tests reliably.
Data exclusions	No data were excluded from the analysis.
Replication	All tests with replicates were successful. Imaging experiments were performed independently 3 times each to ensure reproducibility. ChIP-seq, RNA-seq and Hi-C experiments were undertaken 2-3 times each, as described in the methods. Phase separation assays were performed independently twice.
Randomization	Samples were allocated to groups randomly.
Blinding	All imaging analyses were double-blinded. Investigators were blinded in fertility experiments. Blinding was not relevant in sequencing experiments as it was not relevant to that type of data collection.

Reporting for specific materials, systems and methods

We require information from authors about some types of materials, experimental systems and methods used in many studies. Here, indicate whether each material, system or method listed is relevant to your study. If you are not sure if a list item applies to your research, read the appropriate section before selecting a response.

Materials & experimental systems

n/a	Involved in the study
☐	☒ Antibodies
☒	☐ Eukaryotic cell lines
☒	☐ Palaeontology and archaeology
☒	☐ Animals and other organisms
☒	☐ Clinical data
☒	☐ Dual use research of concern

Methods

n/a	Involved in the study
☐	☒ ChIP-seq
☐	☒ Flow cytometry
☒	☐ MRI-based neuroimaging

Antibodies

Antibodies used	anti-H3K9me2; abcam; ab1220<br>GFP-Trap; ChromoTek; gta-100
Validation	ab1220 - The manufacturer has performed peptide ELISA and Western blots to demonstrate ab1220 recognizes H3K9me2 only and not other methylated lysines in histone tails.<br>gta-100 - Manufacturer has validated the use in anti-GFP ChIP-seq along with ~3000 publications.

ChIP-seq

Data deposition

☒ Confirm that both raw and final processed data have been deposited in a public database such as GEO.

☒ Confirm that you have deposited or provided access to graph files (e.g. BED files) for the called peaks.

Data access links<br>*May remain private before publication.*	https://www.ncbi.nlm.nih.gov/geo/query/acc.cgi?acc=GSE161366
Files in database submission	Native ChIP-seq profiles (IP and inputs) of H2B.8 in sperm (pH2B.8::H2B.8-eGFP) and ectopic H2B.8 (p35S::H2B.8-eGFP), H2B.2 (p35S::H2B.2-eGFP), H2B.8deltaIDR (p35S::H2B.8deltaIDR-eGFP) and H3K9me2 in ectopic H2B.8 (p35S::H2B.8-eGFP) seedlings. Raw data reads (FASTQ format) and normalized tracks (bigwig format) are available along with called peaks (BED format).
Genome browser session<br>(e.g. UCSC)	Bigwig files with normalized ChIP-seq profiles are provided in the GEO submission and can be viewed using IGV.

Methodology

Replicates	Two biological replicates of ChIP-seq libraries were produced for each genotype.
Sequencing depth	ChIP-seq libraries were sequenced with 2 × 38 bp paired end reads to a depth of 30 million reads.
Antibodies	GFP-Trap; ChromoTek; gta-100<br>anti-H3K9me2; abcam; ab1220
Peak calling parameters	Peaks were called using the pipeline as follows. H2B.8 enrichment was calculated over 50 bp windows and those with > 1.2 log2(IP/input) were retained. Windows within 150 bp were merged using BEDtools-2.28.0. Regions were filtered by size, with those < 200 bp removed from analysis. H2B.8 enrichment was then calculated over the new regions, those with < 1.2 log2(IP/input) were discarded. The remaining regions were defined as H2B.8 peaks.
Data quality	Peaks were manually validated in the genome browser to ensure they matched the ChIP-seq profiles.
Software	BEDtools-2.28.0

Flow Cytometry

Plots

Confirm that:

☒ The axis labels state the marker and fluorochrome used (e.g. CD4-FITC).

☒ The axis scales are clearly visible. Include numbers along axes only for bottom left plot of group (a 'group' is an analysis of identical markers).

☒ All plots are contour plots with outliers or pseudocolor plots.

☒ A numerical value for number of cells or percentage (with statistics) is provided.

Methodology

Sample preparation	Sperm and vegetative nuclei were isolated by FACS as described by Borges et al. (2012); sperm cells were isolated by FACS as described by Santos et al. (2017).
Instrument	BD FACSMelody™ cell sorter.
Software	BD FACSChorus™ software version 1.3.
Cell population abundance	Sample purity was assessed by microscopy and was >99%.
Gating strategy	To isolate sperm and vegetative nuclei, events were gated by SYBR Green intensity (FITC-A) and side scatter area (SSC-A) and then by forward scatter area (FSC-A). Nuclei types form two distinct populations owing to their different fluorescence intensities and nuclear sizes. To purify sperm cells from pollen, all events were gated for SYBR Green intensity (FITC-A) and side-scatter (SSC-A) to remove non-cell events. Sperm cells form a clear population, separate from sperm and vegetative nuclei, when gated for SYTOX Orange staining (PE-Cy7 (YG)-A).

☒ Tick this box to confirm that a figure exemplifying the gating strategy is provided in the Supplementary Information.
